# (*E*)-6-Bromo-3-{2-[2-(4-chloro­benzyl­idene)hydrazin­yl]thia­zol-5-yl}-2*H*-chromen-2-one

**DOI:** 10.1107/S1600536811011172

**Published:** 2011-03-31

**Authors:** Afsheen Arshad, Hasnah Osman, Chan Kit Lam, Madhukar Hemamalini, Hoong-Kun Fun

**Affiliations:** a School of Chemical Sciences, Universiti Sains Malaysia, 11800 USM, Penang, Malaysia; b School of Pharmaceutical Sciences, Universiti Sains Malaysia, 11800 USM, Penang, Malaysia; c X-ray Crystallography Unit, School of Physics, Universiti Sains Malaysia, 11800 USM, Penang, Malaysia

## Abstract

In the title compound, C_19_H_11_N_3_O_2_SClBr, the chromene ring system and the thia­zole ring are each approximately planar, with maximum deviations of 0.033 (3) Å and 0.006 (3) Å, respectively. The mol­ecule adopts an *E* configuration about the central C=N double bond. The central thia­zole ring makes dihedral angles of 9.06 (14)° and 12.07 (11)° with the chloro-substituted phenyl ring and the chromene ring, respectively. The mol­ecular structure features a short C—H⋯O contact, which generates an *S*(6) ring motif. The crystal structure is stabilized by inter­molecular N—H⋯O hydrogen bonds, which link the mol­ecules into chains along the *b* axis. π–π stacking inter­actions [centroid-centroid distance = 3.4813 (15) Å] are also present.

## Related literature

For the biological activity and applications of thia­zolyl coumarin derivatives, see: Samsonova *et al.* (2007 ▶); Bullock *et al.* (2009 ▶); Siddiqui *et al.* (2009 ▶); Kalkhambkar *et al.* (2007 ▶); Kamal *et al.* (2009 ▶); Desai *et al.* (2008 ▶). For the synthesis of the title compound, see: Bakkar *et al.* (2003 ▶); Vijesh *et al.* (2010 ▶). For graph-set notation, see: Bernstein *et al.* (1995 ▶).
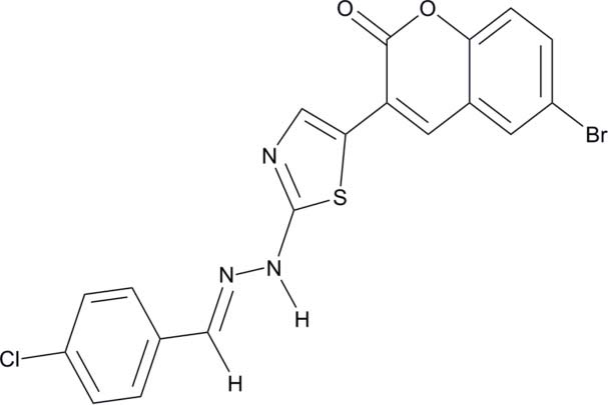


## Experimental

### 

#### Crystal data

C_19_H_11_BrClN_3_O_2_S
*M* *_r_* = 460.73
Monoclinic, 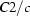

*a* = 30.5837 (7) Å
*b* = 13.6682 (3) Å
*c* = 9.0454 (1) Å
β = 90.161 (2)°
*V* = 3781.18 (13) Å^3^
*Z* = 8
Mo *K*α radiation
μ = 2.45 mm^−1^
*T* = 296 K
0.21 × 0.16 × 0.07 mm

#### Data collection

Bruker SMART APEXII CCD area-detector diffractometer
Absorption correction: multi-scan (*SADABS*; Bruker, 2009 ▶) *T*_min_ = 0.628, *T*_max_ = 0.842
29664 measured reflections
5499 independent reflections
2216 reflections with *I* > 2σ(*I*)
*R*_int_ = 0.058

#### Refinement

*R*[*F* ^2^ > 2σ(*F* ^2^)] = 0.049
*wR*(*F* ^2^) = 0.138
*S* = 0.97
5499 reflections
248 parameters
H atoms treated by a mixture of independent and constrained refinement
Δρ_max_ = 0.31 e Å^−3^
Δρ_min_ = −0.34 e Å^−3^

### 

Data collection: *APEX2* (Bruker, 2009 ▶); cell refinement: *SAINT* (Bruker, 2009 ▶); data reduction: *SAINT*; program(s) used to solve structure: *SHELXTL* (Sheldrick, 2008 ▶); program(s) used to refine structure: *SHELXTL*; molecular graphics: *SHELXTL*; software used to prepare material for publication: *SHELXTL* and *PLATON* (Spek, 2009 ▶).

## Supplementary Material

Crystal structure: contains datablocks global, I. DOI: 10.1107/S1600536811011172/sj5122sup1.cif

Structure factors: contains datablocks I. DOI: 10.1107/S1600536811011172/sj5122Isup2.hkl

Additional supplementary materials:  crystallographic information; 3D view; checkCIF report

## Figures and Tables

**Table 1:** Hydrogen-bond geometry (Å, °)

*D*—H⋯*A*	*D*—H	H⋯*A*	*D*⋯*A*	*D*—H⋯*A*
N2—H1*N*2⋯O2^i^	0.81 (4)	2.16 (4)	2.957 (4)	169 (4)
C11—H11*A*⋯O2	0.93	2.35	2.878 (4)	115

Symmetry code: (i) 
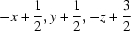
.

**Fig. 1.**
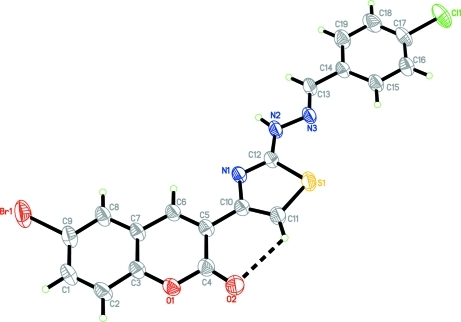
The asymmetric unit of the title compound, showing 30% probability displacement ellipsoids and the atom-numbering scheme. The intramolecular hydrogen bond is shown as a dashed line.

**Fig. 2.**
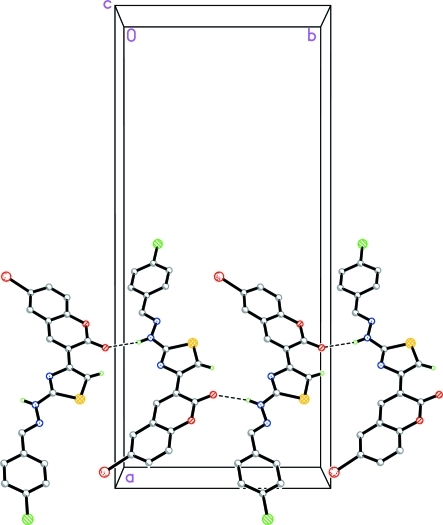
The crystal packing of the title compound (I).

## supplementary crystallographic information

### Comment

Thiazolyl coumarin derivatives are reported to be associated with diverse applications. They have industrial applications as fluorescent probes, laser dyes (Samsonova *et al.*, 2007) and luminescents (Bullock *et al.*, 2009). They also exhibit a variety of biological activities as anticonvulsants (Siddiqui *et al.*, 2009), analgesics, anti-inflammatory (Kalkhambkar *et al.*, 2007) and antimicrobial agents (Kamal *et al.*, 2009; Desai *et al.*, 2008). The title compound is a new derivative of thiazolylcoumarin. We present here its crystal structure.

The molecular structure of the compound, (I), displays a trans configuration with respect to the C13═N3 double bond.  The chromene (O1/C1–C9) ring system and thiazole (S1/N1/C10–C12) ring are approximately planar, with the maximum deviation of 0.033 (3) Å for atom C2 and 0.006 (3)Å for atom C12, respectively. The central thiazole (S1/N1/C10–C12) ring makes dihedral angles of 9.06 (14)° and 12.07 (11)° with the chloro-substituted phenyl (C14–C19) ring and the chromene (O1/C1–C9) ring, respectively.

In the crystal packing (Fig. 2), the molecular structure is stabilized by an intramolecular C—H···O (Table 1) hydrogen bond which generates an *S*(6) (Bernstein *et al.*, 1995) ring motif. Furthermore, the crystal structure is stabilized by intermolecular N—H···O hydrogen bonds which link the molecules into chains parallel to the *b*-axis. π···π [centroid-centroid distance = 3.4813 (15) Å; 1/2-X, 1/2-Y, 2-Z] stacking interactions between the thiazole (S1/N1/C10–C12) and pyran (O1/C3–C7) rings are also observed.

### Experimental

4-chlorobenzylidene thiosemicarbazone (Bakkar *et al.*, 2003) and 6-bromo-3-(2-bromoacetyl)-2*H*-chromen-2-one (Vijesh *et al.*, 2010) were synthesized as reported in the literature. The title compound (I) was obtained by the cyclocondensation of 4-chlorobenzylidene thiosemicarbazone with 6-bromo-3-(2-bromoacetyl)- 2*H*-chromen-2-one.  A solution of 6-bromo-3-(2-bromoacetyl)-2*H*- chromen-2-one (2.5 mmol) and 4-chlorobenzylidene thiosemicarbazone (2.5 mmol) in chloroform-ethanol (2:1) was refluxed for 2 hours at 60°C to yield a dense yellow precipitate. The reaction mixture was cooled in ice bath and basified with ammonia to pH 7–8. The title compound (I) was recrystallized from ethanol-chloroform (1:2) to give yellow block like crystals.

### Refinement

Atom H1N2 was located from a difference Fourier map and refined freely [N–H = 0.81 (4) Å]. The remaining H atoms were positioned geometrically [C–H = 0.93 Å] and were refined using a riding model, with *U*_iso_(H) = 1.2 *U*_eq_(C).   The highest residual electron density peak is located at 1.20 Å from Br1 and the deepest hole 0.91 located at from Br1.

### Crystal data

**Table:**

C_19_H_11_BrClN_3_O_2_S	*F*(000) = 1840
*M**_r_* = 460.73	*D*_x_ = 1.619 Mg m^−^^3^
Monoclinic, *C*2/*c*	Mo *K*α radiation, λ = 0.71073 Å
Hall symbol: -C 2yc	Cell parameters from 3684 reflections
*a* = 30.5837 (7) Å	θ = 2.5–23.1°
*b* = 13.6682 (3) Å	µ = 2.45 mm^−^^1^
*c* = 9.0454 (1) Å	*T* = 296 K
β = 90.161 (2)°	Block, yellow
*V* = 3781.18 (13) Å^3^	0.21 × 0.16 × 0.07 mm
*Z* = 8	

### Data collection

**Table:**

Bruker SMART APEXII CCD area-detector diffractometer	5499 independent reflections
Radiation source: fine-focus sealed tube	2216 reflections with *I* > 2σ(*I*)
graphite	*R*_int_ = 0.058
φ and ω scans	θ_max_ = 30.0°, θ_min_ = 2.5°
Absorption correction: multi-scan (*SADABS*; Bruker, 2009)	*h* = −40→42
*T*_min_ = 0.628, *T*_max_ = 0.842	*k* = −19→19
29664 measured reflections	*l* = −12→12

### Refinement

**Table:**

Refinement on *F*^2^	Primary atom site location: structure-invariant direct methods
Least-squares matrix: full	Secondary atom site location: difference Fourier map
*R*[*F*^2^ > 2σ(*F*^2^)] = 0.049	Hydrogen site location: inferred from neighbouring sites
*wR*(*F*^2^) = 0.138	H atoms treated by a mixture of independent and constrained refinement
*S* = 0.97	*w* = 1/[σ^2^(*F*_o_^2^) + (0.0551*P*)^2^ + 0.960*P*] where *P* = (*F*_o_^2^ + 2*F*_c_^2^)/3
5499 reflections	(Δ/σ)_max_ = 0.001
248 parameters	Δρ_max_ = 0.31 e Å^−^^3^
0 restraints	Δρ_min_ = −0.34 e Å^−^^3^

### Special details

**Table:**

Geometry. All esds (except the esd in the dihedral angle between two l.s. planes) are estimated using the full covariance matrix. The cell esds are taken into account individually in the estimation of esds in distances, angles and torsion angles; correlations between esds in cell parameters are only used when they are defined by crystal symmetry. An approximate (isotropic) treatment of cell esds is used for estimating esds involving l.s. planes.
Refinement. Refinement of F^2^ against ALL reflections. The weighted R-factor wR and goodness of fit S are based on F^2^, conventional R-factors R are based on F, with F set to zero for negative F^2^. The threshold expression of F^2^ > 2sigma(F^2^) is used only for calculating R-factors(gt) etc. and is not relevant to the choice of reflections for refinement. R-factors based on F^2^ are statistically about twice as large as those based on F, and R- factors based on ALL data will be even larger.

### Fractional atomic coordinates and isotropic or equivalent isotropic displacement parameters (Å^2^)

**Table:**

	*x*	*y*	*z*	*U*_iso_*/*U*_eq_	
Br1	0.437955 (16)	0.51731 (4)	1.13651 (6)	0.1287 (3)	
Cl1	−0.00680 (3)	0.31602 (10)	−0.05275 (11)	0.1132 (4)	
S1	0.19723 (3)	0.13873 (7)	0.60268 (9)	0.0712 (3)	
O1	0.33937 (7)	0.13971 (15)	1.0762 (2)	0.0679 (6)	
O2	0.29214 (8)	0.04381 (19)	0.9684 (3)	0.0902 (7)	
N1	0.23951 (7)	0.28268 (18)	0.7199 (2)	0.0575 (6)	
N2	0.18701 (9)	0.3330 (2)	0.5513 (3)	0.0698 (8)	
N3	0.15428 (8)	0.3072 (2)	0.4558 (2)	0.0629 (7)	
C1	0.41549 (11)	0.3221 (3)	1.2104 (4)	0.0778 (10)	
H1A	0.4381	0.3273	1.2790	0.093*	
C2	0.39334 (10)	0.2362 (3)	1.1954 (3)	0.0738 (9)	
H2A	0.4009	0.1825	1.2533	0.089*	
C3	0.35967 (9)	0.2291 (2)	1.0938 (3)	0.0609 (8)	
C4	0.30642 (10)	0.1259 (3)	0.9763 (3)	0.0652 (8)	
C5	0.29156 (9)	0.2090 (2)	0.8891 (3)	0.0556 (7)	
C6	0.31145 (9)	0.2961 (2)	0.9077 (3)	0.0591 (8)	
H6A	0.3018	0.3493	0.8524	0.071*	
C7	0.34686 (9)	0.3096 (2)	1.0094 (3)	0.0601 (8)	
C8	0.36987 (9)	0.3966 (3)	1.0262 (4)	0.0715 (9)	
H8A	0.3620	0.4515	0.9716	0.086*	
C9	0.40440 (10)	0.4013 (3)	1.1240 (4)	0.0766 (10)	
C10	0.25573 (9)	0.1967 (2)	0.7827 (3)	0.0569 (7)	
C11	0.23679 (10)	0.1131 (2)	0.7328 (3)	0.0662 (8)	
H11A	0.2442	0.0505	0.7645	0.079*	
C12	0.20879 (9)	0.2616 (2)	0.6261 (3)	0.0583 (8)	
C13	0.13768 (10)	0.3778 (3)	0.3809 (3)	0.0666 (8)	
H13A	0.1486	0.4408	0.3935	0.080*	
C14	0.10205 (10)	0.3614 (2)	0.2765 (3)	0.0609 (8)	
C15	0.08312 (10)	0.2710 (3)	0.2569 (3)	0.0713 (9)	
H15A	0.0931	0.2183	0.3128	0.086*	
C16	0.04983 (10)	0.2566 (3)	0.1567 (3)	0.0763 (10)	
H16A	0.0377	0.1948	0.1440	0.092*	
C17	0.03487 (10)	0.3341 (3)	0.0762 (3)	0.0767 (10)	
C18	0.05284 (12)	0.4249 (3)	0.0907 (4)	0.0925 (12)	
H18A	0.0426	0.4771	0.0344	0.111*	
C19	0.08655 (11)	0.4378 (3)	0.1909 (4)	0.0846 (10)	
H19A	0.0991	0.4994	0.2010	0.102*	
H1N2	0.1963 (12)	0.388 (3)	0.547 (4)	0.092 (14)*	

### Atomic displacement parameters (Å^2^)

**Table:**

	*U*^11^	*U*^22^	*U*^33^	*U*^12^	*U*^13^	*U*^23^
Br1	0.1122 (4)	0.0915 (4)	0.1821 (5)	−0.0164 (3)	−0.0729 (4)	−0.0212 (3)
Cl1	0.0812 (6)	0.1664 (12)	0.0917 (6)	0.0210 (7)	−0.0463 (5)	−0.0255 (7)
S1	0.0712 (5)	0.0676 (6)	0.0748 (5)	−0.0092 (4)	−0.0221 (4)	−0.0101 (4)
O1	0.0663 (13)	0.0633 (15)	0.0739 (13)	0.0083 (11)	−0.0199 (11)	0.0053 (11)
O2	0.0935 (17)	0.0603 (16)	0.1165 (19)	−0.0022 (14)	−0.0368 (14)	0.0108 (15)
N1	0.0574 (14)	0.0579 (16)	0.0572 (13)	0.0024 (12)	−0.0134 (12)	−0.0054 (12)
N2	0.0731 (18)	0.065 (2)	0.0715 (17)	−0.0067 (16)	−0.0335 (14)	−0.0020 (16)
N3	0.0618 (15)	0.0675 (18)	0.0593 (14)	−0.0034 (13)	−0.0222 (12)	−0.0035 (13)
C1	0.062 (2)	0.092 (3)	0.079 (2)	0.012 (2)	−0.0280 (17)	−0.015 (2)
C2	0.071 (2)	0.078 (2)	0.073 (2)	0.0179 (19)	−0.0248 (17)	0.0020 (19)
C3	0.0561 (17)	0.064 (2)	0.0623 (18)	0.0080 (16)	−0.0080 (15)	−0.0052 (16)
C4	0.0615 (19)	0.059 (2)	0.075 (2)	0.0033 (17)	−0.0123 (16)	0.0000 (18)
C5	0.0544 (16)	0.057 (2)	0.0556 (16)	0.0079 (15)	−0.0070 (13)	−0.0087 (15)
C6	0.0574 (17)	0.0558 (19)	0.0639 (17)	0.0043 (15)	−0.0139 (14)	−0.0037 (15)
C7	0.0523 (16)	0.064 (2)	0.0638 (18)	0.0106 (15)	−0.0140 (14)	−0.0096 (16)
C8	0.0622 (19)	0.063 (2)	0.089 (2)	0.0099 (17)	−0.0253 (17)	−0.0119 (19)
C9	0.066 (2)	0.073 (2)	0.091 (2)	0.0052 (18)	−0.0242 (18)	−0.022 (2)
C10	0.0553 (17)	0.058 (2)	0.0577 (17)	0.0031 (15)	−0.0051 (14)	−0.0033 (15)
C11	0.0664 (19)	0.058 (2)	0.074 (2)	0.0010 (16)	−0.0174 (16)	−0.0036 (17)
C12	0.0579 (17)	0.064 (2)	0.0535 (16)	0.0003 (15)	−0.0114 (14)	−0.0064 (15)
C13	0.0686 (19)	0.068 (2)	0.0634 (18)	−0.0012 (17)	−0.0199 (16)	−0.0117 (17)
C14	0.0605 (18)	0.063 (2)	0.0592 (17)	0.0073 (16)	−0.0150 (15)	−0.0078 (16)
C15	0.0673 (19)	0.078 (2)	0.0688 (19)	0.0023 (18)	−0.0228 (16)	−0.0028 (18)
C16	0.066 (2)	0.085 (3)	0.078 (2)	−0.0002 (19)	−0.0202 (17)	−0.011 (2)
C17	0.061 (2)	0.106 (3)	0.0624 (19)	0.017 (2)	−0.0232 (16)	−0.015 (2)
C18	0.095 (3)	0.095 (3)	0.088 (3)	0.025 (2)	−0.037 (2)	0.005 (2)
C19	0.089 (2)	0.074 (2)	0.090 (2)	0.009 (2)	−0.032 (2)	−0.004 (2)

### Geometric parameters (Å, °)

**Table:**

Br1—C9	1.892 (3)	C5—C10	1.466 (4)
Cl1—C17	1.743 (3)	C6—C7	1.431 (4)
S1—C11	1.721 (3)	C6—H6A	0.9300
S1—C12	1.728 (3)	C7—C8	1.390 (4)
O1—C4	1.365 (4)	C8—C9	1.377 (4)
O1—C3	1.379 (4)	C8—H8A	0.9300
O2—C4	1.206 (4)	C10—C11	1.359 (4)
N1—C12	1.297 (3)	C11—H11A	0.9300
N1—C10	1.395 (4)	C13—C14	1.458 (4)
N2—C12	1.361 (4)	C13—H13A	0.9300
N2—N3	1.366 (3)	C14—C15	1.376 (4)
N2—H1N2	0.81 (4)	C14—C19	1.383 (4)
N3—C13	1.283 (4)	C15—C16	1.376 (4)
C1—C2	1.363 (5)	C15—H15A	0.9300
C1—C9	1.377 (5)	C16—C17	1.364 (5)
C1—H1A	0.9300	C16—H16A	0.9300
C2—C3	1.382 (4)	C17—C18	1.363 (5)
C2—H2A	0.9300	C18—C19	1.382 (5)
C3—C7	1.395 (4)	C18—H18A	0.9300
C4—C5	1.455 (4)	C19—H19A	0.9300
C5—C6	1.347 (4)		
			
C11—S1—C12	88.34 (15)	C1—C9—Br1	119.5 (2)
C4—O1—C3	122.0 (2)	C8—C9—Br1	119.5 (3)
C12—N1—C10	109.6 (2)	C11—C10—N1	115.0 (2)
C12—N2—N3	119.1 (3)	C11—C10—C5	129.2 (3)
C12—N2—H1N2	122 (3)	N1—C10—C5	115.8 (3)
N3—N2—H1N2	118 (3)	C10—C11—S1	110.7 (2)
C13—N3—N2	115.4 (3)	C10—C11—H11A	124.6
C2—C1—C9	120.0 (3)	S1—C11—H11A	124.6
C2—C1—H1A	120.0	N1—C12—N2	121.2 (3)
C9—C1—H1A	120.0	N1—C12—S1	116.4 (2)
C1—C2—C3	119.7 (3)	N2—C12—S1	122.4 (2)
C1—C2—H2A	120.1	N3—C13—C14	121.3 (3)
C3—C2—H2A	120.1	N3—C13—H13A	119.3
O1—C3—C2	118.3 (3)	C14—C13—H13A	119.3
O1—C3—C7	120.6 (2)	C15—C14—C19	117.6 (3)
C2—C3—C7	121.1 (3)	C15—C14—C13	122.4 (3)
O2—C4—O1	115.7 (3)	C19—C14—C13	120.0 (3)
O2—C4—C5	125.6 (3)	C16—C15—C14	121.6 (3)
O1—C4—C5	118.7 (3)	C16—C15—H15A	119.2
C6—C5—C4	118.8 (3)	C14—C15—H15A	119.2
C6—C5—C10	121.3 (3)	C17—C16—C15	119.2 (3)
C4—C5—C10	119.9 (3)	C17—C16—H16A	120.4
C5—C6—C7	122.3 (3)	C15—C16—H16A	120.4
C5—C6—H6A	118.8	C18—C17—C16	121.4 (3)
C7—C6—H6A	118.8	C18—C17—Cl1	119.2 (3)
C8—C7—C3	118.3 (3)	C16—C17—Cl1	119.4 (3)
C8—C7—C6	124.2 (3)	C17—C18—C19	118.6 (4)
C3—C7—C6	117.5 (3)	C17—C18—H18A	120.7
C9—C8—C7	119.8 (3)	C19—C18—H18A	120.7
C9—C8—H8A	120.1	C18—C19—C14	121.6 (4)
C7—C8—H8A	120.1	C18—C19—H19A	119.2
C1—C9—C8	121.0 (3)	C14—C19—H19A	119.2
			
C12—N2—N3—C13	−174.7 (3)	C12—N1—C10—C5	178.7 (2)
C9—C1—C2—C3	−0.3 (5)	C6—C5—C10—C11	168.5 (3)
C4—O1—C3—C2	−178.7 (3)	C4—C5—C10—C11	−11.1 (5)
C4—O1—C3—C7	0.3 (4)	C6—C5—C10—N1	−9.3 (4)
C1—C2—C3—O1	176.8 (3)	C4—C5—C10—N1	171.1 (3)
C1—C2—C3—C7	−2.2 (5)	N1—C10—C11—S1	0.1 (3)
C3—O1—C4—O2	178.1 (3)	C5—C10—C11—S1	−177.7 (2)
C3—O1—C4—C5	−1.8 (4)	C12—S1—C11—C10	−0.5 (2)
O2—C4—C5—C6	−178.4 (3)	C10—N1—C12—N2	178.9 (3)
O1—C4—C5—C6	1.4 (4)	C10—N1—C12—S1	−1.0 (3)
O2—C4—C5—C10	1.2 (5)	N3—N2—C12—N1	−178.7 (3)
O1—C4—C5—C10	−179.0 (3)	N3—N2—C12—S1	1.2 (4)
C4—C5—C6—C7	0.4 (4)	C11—S1—C12—N1	0.9 (2)
C10—C5—C6—C7	−179.2 (3)	C11—S1—C12—N2	−179.0 (3)
O1—C3—C7—C8	−176.8 (3)	N2—N3—C13—C14	−179.1 (3)
C2—C3—C7—C8	2.2 (4)	N3—C13—C14—C15	2.8 (5)
O1—C3—C7—C6	1.5 (4)	N3—C13—C14—C19	−176.2 (3)
C2—C3—C7—C6	−179.5 (3)	C19—C14—C15—C16	−0.3 (5)
C5—C6—C7—C8	176.3 (3)	C13—C14—C15—C16	−179.3 (3)
C5—C6—C7—C3	−1.9 (4)	C14—C15—C16—C17	−0.8 (5)
C3—C7—C8—C9	0.3 (5)	C15—C16—C17—C18	1.4 (5)
C6—C7—C8—C9	−177.9 (3)	C15—C16—C17—Cl1	179.1 (2)
C2—C1—C9—C8	2.8 (5)	C16—C17—C18—C19	−0.8 (6)
C2—C1—C9—Br1	−175.8 (3)	Cl1—C17—C18—C19	−178.5 (3)
C7—C8—C9—C1	−2.8 (5)	C17—C18—C19—C14	−0.4 (6)
C7—C8—C9—Br1	175.8 (2)	C15—C14—C19—C18	1.0 (5)
C12—N1—C10—C11	0.6 (4)	C13—C14—C19—C18	180.0 (3)

### Hydrogen-bond geometry (Å, °)

**Table:**

*D*—H···*A*	*D*—H	H···*A*	*D*···*A*	*D*—H···*A*
N2—H1N2···O2^i^	0.81 (4)	2.16 (4)	2.957 (4)	169 (4)
C11—H11A···O2	0.93	2.35	2.878 (4)	115

Symmetry codes: (i) −*x*+1/2, *y*+1/2, −*z*+3/2.
